# Establishment and validation of a logistic regression model for prediction of septic shock severity in children

**DOI:** 10.1186/s41065-021-00206-9

**Published:** 2021-11-12

**Authors:** Yujie Han, Lili Kang, Xianghong Liu, Yuanhua Zhuang, Xiao Chen, Xiaoying Li

**Affiliations:** Department of Neonatal, Qilu Children’s Hospital of Shandong University, No. 23976, Huaiyin District, Jinan City, 250022 Shandong People’s Republic of China

**Keywords:** Septic shock, Logistic regression model, Survival, Systemic lupus erythematosus pathway, Limonene and pinene degradation pathway

## Abstract

**Background:**

Septic shock is the most severe complication of sepsis, and is a major cause of childhood mortality, constituting a heavy public health burden.

**Methods:**

We analyzed the gene expression profiles of septic shock and control samples from the Gene Expression Omnibus (GEO). Four differentially expressed genes (DEGs) from survivor and control groups, non-survivor and control groups, and survivor and non-survivor groups were selected. We used data about these genes to establish a logistic regression model for predicting the survival of septic shock patients.

**Results:**

Leave-one-out cross validation and receiver operating characteristic (ROC) analysis indicated that this model had good accuracy. Differential expression and Gene Set Enrichment Analysis (GSEA) between septic shock patients stratified by prediction score indicated that the systemic lupus erythematosus pathway was activated, while the limonene and pinene degradation pathways were inactivated in the high score group.

**Conclusions:**

Our study provides a novel approach for the prediction of the severity of pathology in septic shock patients, which are significant for personalized treatment as well as prognostic assessment.

**Supplementary Information:**

The online version contains supplementary material available at 10.1186/s41065-021-00206-9.

## Introduction

Sepsis is an acute organ dysfunction that is secondary to infection [1]. It is a major cause of death in patients with complex conditions including humoral and cellular reactions, inflammatory and anti-inflammatory issues, and circulatory problems [2–4]. The most severe complication of sepsis, septic shock, has a mortality of 20–35% [5]. Septic shock is characterized by hemodynamic alterations including hypovolemia, decrease in vascular tone, and myocardial depression associated with organ dysfunction [6]. Therapies consist of inhibition of bacterial antioxidant mechanism and biofilm formation, antimicrobials, hyperbaric oxygen and ozone therapies, bacteriophage therapy, etc. [7], and the treatment depends upon the extent of disease progression.

The exploration of prognostic factors for septic shock will be valuable for the assessment of patients and decisions about interventions and treatment adjustment [8]. Several prognostic factors for sepsis and septic shock have been investigated. It has been previously reported that the short-term prognosis of septic shock is influenced by multiorgan failure, concordance of empiric antibiotic treatment with sensitivity testing in vitro, presence of more than two comorbidities, and Karnofsky score, while higher organ failure score, relapse of hematologic disease, Karnofsky score and resistance to treatment are important prognostic factors for long-term prognosis [9]. Procalcitonin (PCT) has been used as an early diagnostic marker for sepsis [10]. However, individual determination of PCT produces variable results, and may not be an effective diagnostic approach [11, 12]. Serial determinations of PCT have been shown to independently predict the mortality of severe sepsis [13], but this analysis is complex and time consuming. Circulating histones have been put forward as potential markers for sepsis evolution, but no consistent conclusions have been drawn on the specific concentration of each histone for sepsis monitor [14]. Yan HP et al. suggested that plasma mtDNA might be candidate biomarker for the prognosis of sepsis, but this study lacked serial measurement of mtDNA levels [15].

As the biological processes involved in septic shock are complex, it is proposed that the use of multibiomarkers for the stratification of septic shock may meet both research and clinical needs. In a previous study, 15 candidate biomarkers—*CCL3*, *CCL4*, *ELA2*, *FGL2*, *GZMB*, *HSPA1B*, *IL1A*, *IL8*, *LCN2*, *LTF*, *MMP8*, *ORM1*, *RETN*, *SULF2*, and *THBS*—were selected using a genome-wide expression database [16, 17]. Through integrative bioinformatic methods, Rosier F et al. screened out some genes related to the death of septic shock patients, and demonstrated that the genetic variation in *CISH* resulted in elevated death risk of patients with sepsis [5]. A total of six genes, including *CREBBP*, *WDR82*, *NCOA1*, *ASH1L*, *TPR*, and *SF1*, were identified as prognosis-related genes in patients with septic shock via comprehensively analyzing the gene expression spectrum [18]. *OLFM4* polymorphisms were found to be able to anticipate the clinical outcome of septic shock patients after major surgery [19]. In our study, a logistic regression model was established using data from four vital genes: *G0S2*, *CTSD*, *PRUNE2*, and *SLC22A4*, to predict the severity of septic shock in children, using the analysis of differentially expressed genes (DEGs) among the survivors, non-survivors and controls. The model presents encouraging predictive value in evaluating severity and lethality of children septic shock, which should be helpful for the disease assessment and treatment management.

## Materials and methods

### Study population

The gene expression profiles of 130 whole blood samples were obtained from Gene Expression Omnibus (GEO) (https://www.ncbi.nlm.nih.gov/geo/, Accession number: GSE26440), including 98 children with septic shock and 32 controls with age from 0 to 10.9 and median age of 2. The 98 septic shock patients consisted of 81 survivors and 17 non-survivors. Microarray hybridization was carried out using the Human Genome U133 Plus 2.0 GeneChip (Affymetrix, Santa Clara, CA, USA).

The dataset GSE26440 consisted of the grouping information of groups A, B, and C, which could be used directly. Compared with groups B and C, the patients in group A were younger, with higher disease severity and mortality rate.

### Differential expression analysis

Background correction and normalization of the data were carried out with *Affy* Bioconductor package. The differentially expressed genes (DEGs) were analyzed using limma R package. *P-*value < 0.05 and |log2FC| > 1 (FC: Fold Change) were set as thresholds for significantly differential expression.

### Candidate genes selection and logistic regression analysis

The DEGs between survivor and control groups, non-survivor and control groups, survivor and non-survivor groups were analyzed to obtain their intersection. Pearson correlation was conducted to calculate the correlation between two genes, and genes with Pearson correlation coefficient larger than 0.8 were excluded.

The logistic regression model was established to predict survival probability using stepwise regression method, with the selected genes as independent variables, and survival/death as dependent variables. Leave-one-out cross validation was performed to evaluate the accuracy of the model. Receiver operating characteristic (ROC) curve was used to assess the discrimination of the model and the optimal cutoff point was obtained.

### Function enrichment analysis

The blood samples from patients with septic shock were evaluated by logistic regression model to obtain the prediction scores. According to the prediction scores, septic shock samples were divided into high score and low score groups. Gene Set Enrichment Analysis (GSEA) of the DEGs between two groups was carried out to select the significantly enriched Kyoto Encyclopedia of Genes and Genomes (KEGG) pathways.

## Results

### DEGs between survivor and control, non-survivor and control, survivor and non-survivor groups

**Fig. 1:**
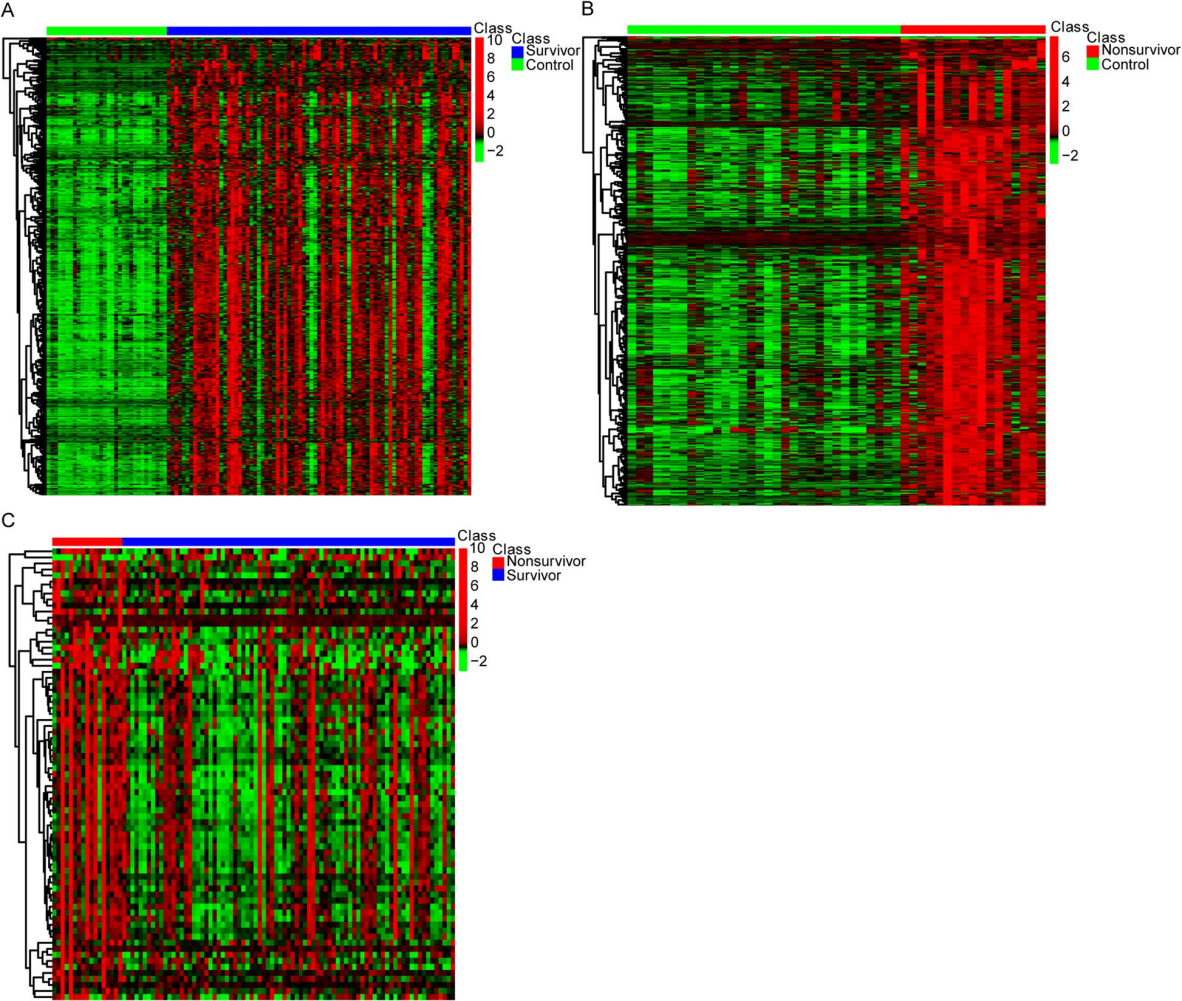
Expression heatmaps of the DEGs in survivor and control, non-survivor and control, survivor and non-survivor groups. **A** Expression heatmap of the DEGs between survivor and control groups. **B** Expression heatmap of the DEGs between non-survivor and control groups. **C** Expression heatmap of the DEGs between survivor and non-survivor groups. DEG: differentially expressed genes

**Fig. 2:**
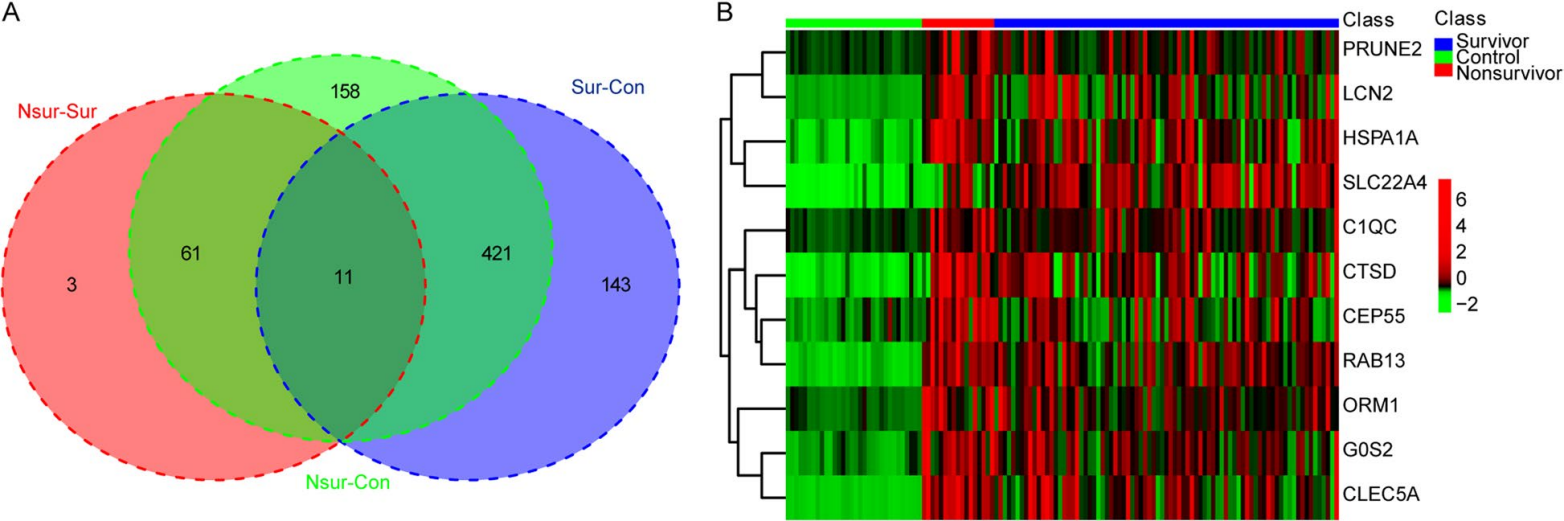
Common DEGs in survivor and control, non-survivor and control, survivor and non-survivor groups. **A** There were 575 DEGs between survivor and control groups, 651 DEGs between non-survivor and control groups, 75 DEGs between survivor and non-survivor groups, and 11 common DEGs among the three groups. **B** Expression heatmap of the 11 common DEGs (*PRUNE2*, *LCN2*, *HSPA1A*, *SLC22A4*, *C1QC*, *CTSD*, *CEP55*, *RAB13*, *ORM1*, *G0S2*, and *CLEC5A*)*.* DEG: differentially expressed genes

We analyzed the DEGs between survivor and control, non-survivor and control, survivor and non-survivor groups, and obtained 575 (Fig. 1A), 651 (Fig. 1B), 75 (Fig. 1C) DEGs respectively. The 11 common DEGs of the three groups were as follows: *PRUNE2*, *LCN2*, *HSPA1A*, *SLC22A4*, *C1QC*, *CTSD*, *CEP55*, *RAB13*, *ORM1*, *G0S2*, and *CLEC5A* (Fig. 2).

### Establishment of logistic regression model

**Fig. 3:**
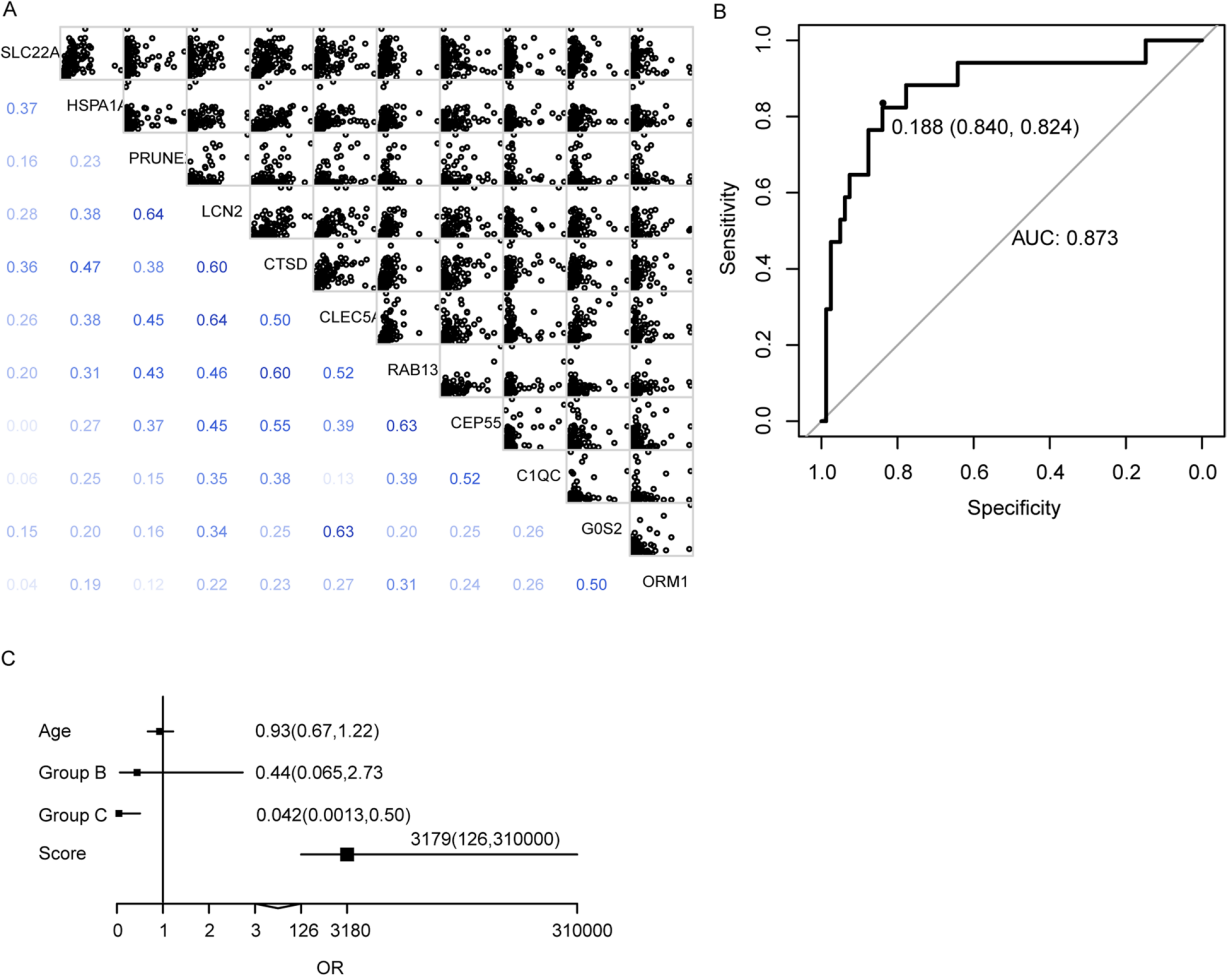
Correlation analysis of the common genes and evaluation of the established logistic regression model. **A** Correlation analysis of the 11 common genes in survivor and control, non-survivor and control, survivor and non-survivor groups. **B** ROC curve showed 0.188 was the optimal cutoff point for survival of septic shock patients, and the AUC was 0.873. **C** Logistic regression analysis of prediction score, age, grouping, survival/death indicated age was not a risk factor for survival (OR: 0.93, 95% CI: 0.67-1.22), and the prediction score of our model could predict the survival accurately (OR: 3197, 95% CI: 126-310000). For the subclasses classified in previous study, the mortality of patients in group C was significantly lower than that in group A (OR: 0.042, 95% CI: 0.0013-0.5), while no significant difference in survival was observed between groups B and A (OR: 0.44, 95% CI: 0.065-2.73). ROC: receiver operating characteristic; AUC: area under ROC curve; OR: odd ratio

Correlation analysis of the 11 genes indicated the Pearson correlation coefficients of them were all less than 0.8 (Fig. 3A), which could be used for logistic regression model. Four genes *G0S2* (*p* = 0.00281), *CTSD* (*p* = 0.01326), *PRUNE2* (*p* = 0.03506), *SLC22A4* (*p* = 0.00615) were selected after stepwise logistic regression analysis for the construction of the predictive model. All samples in the dataset were assigned a risk score which represents death risk through the predictive model based on their expression levels of the four genes. Leave-one-out analysis of the risk score cross validation showed the area under ROC curve (AUC) was 0.873 and the optimal cutoff value of ROC was 0.188 (0.840, 0.824) (Fig. 3B). In addition, samples in the original study of GSE26440 dataset [20] were classified into three subgroups which named group A, group B, and group C through unsupervised hierarchical clustering based on the empiric, discovery-oriented gene expression. Here, the independence of prediction score, age, and grouping for survival/death prediction was investigated by logistic regression analysis with prediction score, age, and grouping as independent variables and survival/death as responsive variables. Figure 3C suggested age was not a risk factor for survival (OR: 0.93, 95% CI: 0.67-1.22); for the subclasses classified in previous study, the mortality of patients in group C was significantly lower than that in group A (OR: 0.042, 95% CI: 0.0013-0.5) and no significant difference in survival was observed between groups B and A (OR: 0.44, 95% CI: 0.065-2.73) [20]. The prediction score was proved to be an accurate indicator for survival (OR: 3179, 95% CI: 126-310000).

### Systemic lupus erythematosus pathway was up-regulated while limonene and pinene degradation pathway was down-regulated in high prediction score group

**Fig. 4:**
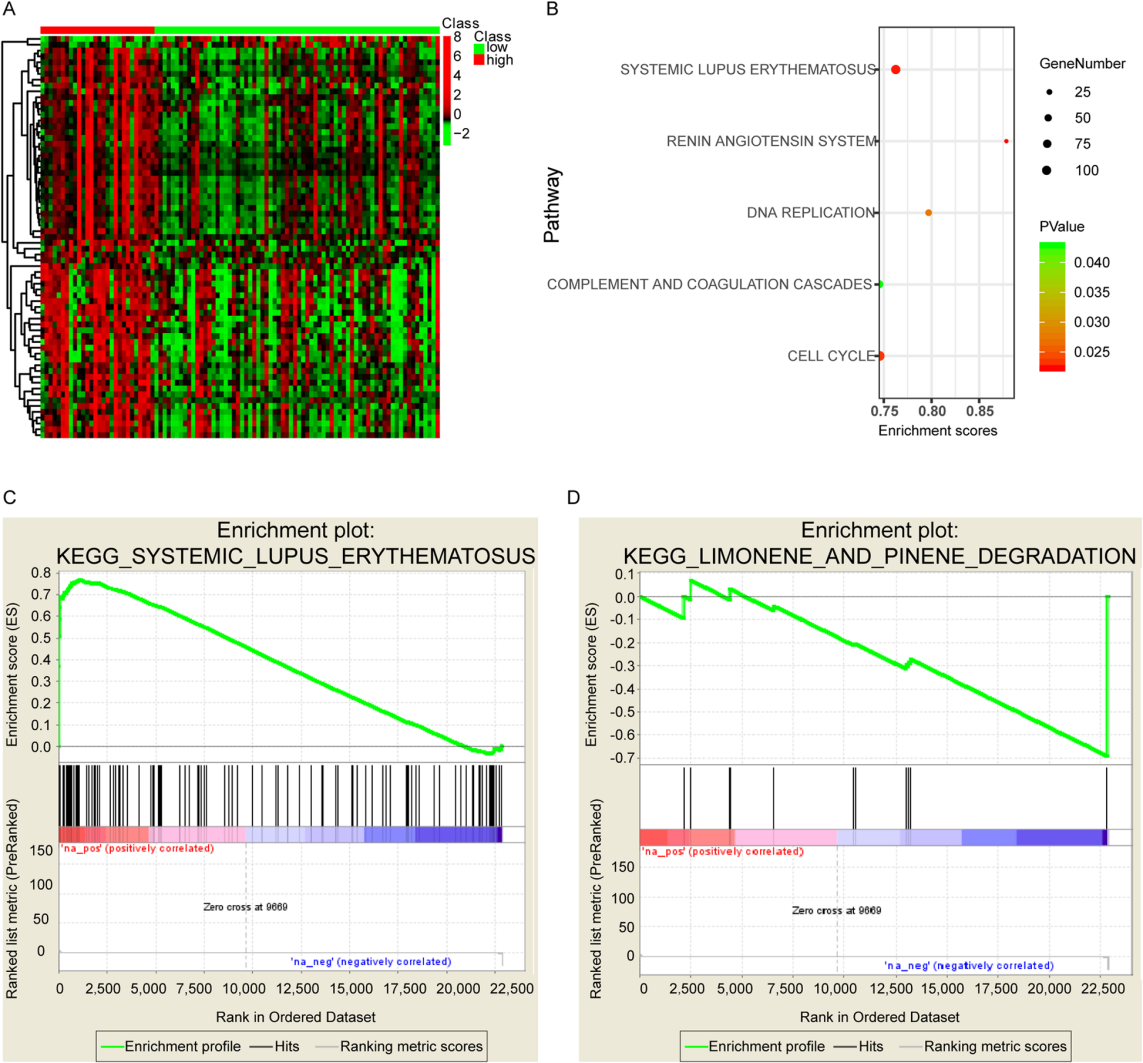
Systemic lupus erythematosus pathway was significantly up-regulated while limonene and pinene degradation pathway was significantly down-regulated in high-prediction score group. **A** Expression heatmap of the 69 DEGs between high score and low score groups. **B** Up-regulation of 5 signal pathways were observed in high prediction score group. **C** Systemic lupus erythematosus pathway was significantly up-regulated in high prediction score group. **D** Limonene and pinene degradation pathway was significantly down-regulated in high prediction score group. DEG: differentially expressed genes

The septic shock samples were divided into high score and low score groups based on the prediction scores, with mean value of the prediction scores as threshold. There were 69 DEGs (Table S1) between high score and low score groups, with the expression heatmap shown in Fig. 4A. GSEA revealed 5 signal pathways were significantly up-regulated in high prediction score group, including systemic lupus erythematosus, cell cycle, complement and coagulation cascades, DNA replication, renin angiotensin system (RAS) pathways and the up-regulation of systemic lupus erythematosus pathway was the most obvious (Fig. 4B, C), while the limonene and pinene degradation pathway was significantly down-regulated (Fig. 4D).

## Discussion

Septic shock is a significant cause of childhood morbidity and mortality all over the world, and generates a heavy public health burden [21, 22]. Septic shock remains the fifth most important cause of years of productive life lost by premature mortality [22]. Septic shock syndrome has multiple causes, and is a major inducer of clinical failure involving the inflammatory or immune systems [23]. A single therapy that targets the inflammatory or immune system may not be valid for a heterogeneous population of patients with septic shock. A classification of septic shock patients will therefore be beneficial for the development of personalized treatments, as well as for prognostic assessment [20].

In our study, the analysis of DEGs between survivor and control groups, non-survivor and control groups, and survivor and non-survivor groups was performed, and 11 common genes were identified. After logistic regression analysis, four genes—*G0S2*, *CTSD*, *PRUNE2*, and *SLC22A4*—were selected with which to establish a model for predicting the severity of septic shock in patients. Leave-one-out cross validation and ROC analysis indicated that the logistic regression model had high accuracy in predicting the survival of septic shock patients. Additionally, samples for gene expression profiling are blood from septic shock patients, which would make the model possible to serve as a noninvasive means for survival evaluation.

Among the four genes, *CTSD* has been proved to be associated with septic shock. It has been shown that TGase 2 could protect liver from the septic shock induced by TNF-α through decreasing *CTSD* expression level [24]. Moreover, compared with untreated septic shock cats, the septic shock cats treated by anisodamine, an alkaloid used for septic shock treatment, had decreased cathepsin D (encoded by *CTSD*) activity [25]. Although there are no direct evidences on the relationship between *G0S2*, *PRUNE2*, *SLC22A4* and septic shock or sepsis, several researches indicated their indirect association. *PRUNE2* is considered as a regulator of Rho signaling [26]. It is known that Rho could interact with Rho kinase, which has the therapeutic effects on alleviations of inflammation and coagulation dysfunction in sepsis and is recognized as the promising therapeutic target [27, 28]. Hence, *PRUNE2* might participate in sepsis through regulating Rho signaling and Rho kinase. *SLC22A4*, also known as *OCTN1*, is regulated by several factors including inflammatory cytokines, and related to inflammatory diseases [29]. It is suggested that inflammatory cytokines play a pivotal role in the organ damage during inflammatory disease of septic shock [30]. Thus, we inferred that *SLC22A4* might be involved in septic shock with the interaction of inflammatory cytokines. A previous study showed that overexpression of *Bcl-2* in septic mice could improve the mortality with anti-apoptosis effects on the intestinal epithelial cell [31]. As we know, *G0S2* inhibits the anti-apoptosis function of *Bcl-2* by suppressing the formation of Bcl-2/Bax heterodimeric complexes [32]. These results indicated that *G0S2* might play a pro-apoptosis role in the intestinal epithelium of sepsis, contributing to a high mortality. Taken together, the up-regulation of these four genes may play a role in immunosuppression of sepsis by interfering with downstream signal pathways or macromolecular complexes formation.

The development of a system of classification of septic shock patients could increase the efficiency of hemodynamic management [33], and numerous efforts have been made to search for biomarkers for septic shock classification. Biomarkers identified to date include IL-8 and CCL4 [17]. However, as septic shock is a complex and heterogeneous condition, the classification made using these biomarkers has been shown to be simplistic, with limited sensitivity, specificity, and positive predictive value [34–36]. A potential alternative to this approach is the use of genome-wide expression profiles. The significant expression of genes that are grouped in a score could improve the validity of each individual to predict the survival. Wong HR et al. identified three putative subclasses: A, B, and C, by analyzing DEGs between septic shock patients and controls. The data collected by Wong HR et al. in infants are well done and very informative in absence of major comorbidities, as their transposition to the adults may be limited by the chronic diseases and chronic treatment, or comorbidities [20]. Patients in subclass A showed more severe illness and higher mortality rates at a younger age than patients in subclasses B and C [20]. In contrast, the model we developed to predict the survival of septic shock patients takes into account the difference between survivors and non-survivors.

We predicted the scores of septic shock patients using our model, and divided them into high-scoring and low-scoring groups, based on the prediction scores. A total of 69 DEGs were observed between the two groups, and the DEGs were significantly enriched in six signal pathways. Among them, the pathways such as systemic lupus erythematosus and RAS were significantly up-regulated, while the limonene and pinene degradation pathway was significantly down-regulated.

Systemic lupus erythematosus is a chronic autoimmune disorder characterized by the production of autoantibodies against nuclear and cytoplasmic antigens [37]. Infection caused by common opportunistic agents is the major reason for morbidity and mortality in immunocompromised systemic lupus erythematosus patients [38–40]. Sepsis is one of the leading causes for death of systemic lupus erythematosus patients [41]. A patient suffering from systemic lupus erythematosus with septic shock caused by a virus has been reported [42]. A high incidence of *Salmonella* infections, which may develop sepsis, has been reported in systemic lupus erythematosus patients [40]. Immunosuppression is considered to be the pivotal host response in sepsis, contributing to the susceptibility to infection of systemic lupus erythematosus patients, and is recognized as one of the vital variables for systemic lupus erythematosus [43–45]. It is hypothesized that the immunosuppression caused by septic shock may lead to the up-regulation of the systemic lupus erythematosus pathway.

As a crucial neuroendocrine system, RAS has been a hotspot in the research area of sepsis. The angiotensin I transforms into angiotensin II when the blood pressure is reduced; Angiotensin II, the main active peptide in RAS, plays a key role in increasing the blood pressure with the combination of ATR [46]. Angiotensin II probably contributes to the aggravation of inflammatory reaction by enhancing the chemokines and proinflammatory cell factors synthesis [47], while sepsis is characterized by severe inflammatory reaction [48]. Tamion F et al. showed that RAS was implicated in the development of sepsis, and sepsis might be related to elevated RAS expression levels [49]. This may explain why the septic shock patients with high prediction score has significantly up-regulated RAS pathway in our study.

Limonene is a natural component in citrus fruits which exhibits anti-inflammatory function and could alleviate inflammation by targeting A_2A_ receptors [50]. α-pinene is a compound in the oils of coniferous trees as well as a main ingredient of volatiles that are extracted from various types of trees [51]. It has multiple biological functions including the an-inflammatory effect. Kim DS et al. found that α-pinene could inhibit the inflammatory response and is a promising alternative for the treatment of inflammation [52]. Considering the anti-inflammatory effects of limonene and pinene, it is assumed that the down-regulated limonene and pinene degradation pathway in the high-scoring group may increase the levels of limonene and pinene to antagonize the inflammatory response caused by septic shock. However, the underlying mechanism still warrants further investigation.

In conclusion, we established a logistic regression model for predicting the survival of pediatric septic shock patients, and validated its accuracy. In septic shock patients stratified by prediction score, the systemic lupus erythematosus pathway was activated, while the limonene and pinene degradation pathway was inactivated in the high-prediction score group. Our research provides a novel approach to the assessment of the survival status of septic shock patients, and can be valuable for the development of personalized treatment.

## Supplementary Information

**Additional file 1: Table S1**. DEGs between high and low risk score sepsis samples.

## Data Availability

All data generated or analyzed during this study are included in this published article.
