# Supplementary material for: Establishment and validation of a logistic regression model for prediction of septic shock severity in children
Source: Hereditas. 2021 Nov 12;158:45. doi: 10.1186/s41065-021-00206-9 (PMC8588704; doi:10.1186/s41065-021-00206-9)
Supplement: Supplementary file 1 — Additional file 1: Table S1. DEGs between high and low risk score sepsis samples. [file 41065_2021_206_MOESM1_ESM.docx]

Table S1. DEGs between high and low risk score sepsis samples.

| Gene | logFC | AveExpr | t | P.Value | adj.P.Val | B |
| --- | --- | --- | --- | --- | --- | --- |
| GPI | 1.4168 | 1.708429 | 5.780915 | 8.83E-08 | 0.000673 | 7.543216 |
| CLEC5A | 16.20275 | 15.59143 | 5.818983 | 7.46E-08 | 0.000673 | 7.696766 |
| BPI | 4.714871 | 4.813497 | 5.580896 | 2.13E-07 | 0.001217 | 6.744113 |
| LCN2 | 33.30514 | 27.79483 | 5.474151 | 3.38E-07 | 0.00129 | 6.323189 |
| PRC1 | 2.468736 | 2.399582 | 5.277861 | 7.84E-07 | 0.002243 | 5.559898 |
| CTSD | 1.591686 | 2.896796 | 5.12848 | 1.47E-06 | 0.002591 | 4.98886 |
| HSPA1A | 2.410481 | 3.662799 | 5.107181 | 1.61E-06 | 0.00263 | 4.908164 |
| PKM | 1.074914 | 2.23949 | 4.954381 | 3.03E-06 | 0.003468 | 4.334757 |
| PRUNE2 | 2.504336 | 2.466867 | 4.923199 | 3.44E-06 | 0.003494 | 4.218953 |
| TCN1 | 13.51196 | 12.97667 | 4.925714 | 3.41E-06 | 0.003494 | 4.228278 |
| LTF | 33.10376 | 30.5117 | 4.918487 | 3.51E-06 | 0.003494 | 4.201493 |
| NUSAP1 | 1.439721 | 1.842128 | 4.795182 | 5.80E-06 | 0.003993 | 3.747996 |
| CEP55 | 3.515736 | 3.722296 | 4.70145 | 8.45E-06 | 0.004603 | 3.407847 |
| KIF20A | 1.055743 | 1.785327 | 4.651265 | 1.03E-05 | 0.005095 | 3.227405 |
| RETN | 25.80299 | 27.6203 | 4.66277 | 9.86E-06 | 0.005095 | 3.268666 |
| TOP2A | 2.070248 | 2.245395 | 4.63562 | 1.10E-05 | 0.005127 | 3.171394 |
| FOXM1 | 1.055586 | 1.71751 | 4.563376 | 1.46E-05 | 0.005568 | 2.914269 |
| KIF4A | 1.259757 | 1.944173 | 4.495773 | 1.90E-05 | 0.005961 | 2.675957 |
| CDC20 | 1.369586 | 1.841653 | 4.47146 | 2.09E-05 | 0.006073 | 2.590802 |
| CEACAM8 | 48.86501 | 36.6991 | 4.393973 | 2.82E-05 | 0.007681 | 2.321383 |
| SHCBP1 | 2.14485 | 2.130286 | 4.352161 | 3.31E-05 | 0.008414 | 2.177273 |
| KIF11 | 1.990064 | 2.147133 | 4.312644 | 3.85E-05 | 0.009267 | 2.041903 |
| TPX2 | 1.270593 | 1.948612 | 4.275054 | 4.44E-05 | 0.009315 | 1.913891 |
| GBA | 1.271093 | 2.937827 | 4.288062 | 4.22E-05 | 0.009315 | 1.958108 |
| G0S2 | 4.8039 | 5.3995 | 4.276611 | 4.41E-05 | 0.009315 | 1.919179 |
| GPR84 | 10.56614 | 14.5453 | 4.243748 | 4.99E-05 | 0.010292 | 1.80785 |
| RAB13 | 2.515807 | 3.947673 | 4.229486 | 5.27E-05 | 0.010667 | 1.759713 |
| SPC25 | 1.315529 | 1.638194 | 4.181396 | 6.31E-05 | 0.012025 | 1.598203 |
| RGL1 | 1.226721 | 1.935592 | 4.168213 | 6.62E-05 | 0.012126 | 1.554147 |
| DTL | 2.144636 | 2.337332 | 4.147393 | 7.16E-05 | 0.012695 | 1.484762 |
| TYMS | 1.07374 | 1.55234 | 4.141942 | 7.30E-05 | 0.012855 | 1.466633 |
| HIP1 | 1.426389 | 3.067615 | 4.12327 | 7.83E-05 | 0.013095 | 1.404665 |
| UHRF1 | 2.592186 | 2.550582 | 4.125756 | 7.75E-05 | 0.013095 | 1.412903 |
| RRM2 | 3.276321 | 3.02227 | 4.125665 | 7.76E-05 | 0.013095 | 1.412601 |
| PGLYRP1 | 5.345364 | 7.148561 | 4.088648 | 8.89E-05 | 0.014033 | 1.290266 |
| MMP8 | 175.4009 | 201.1904 | 4.090103 | 8.85E-05 | 0.014033 | 1.29506 |
| BUB1B | 1.689807 | 1.832173 | 4.070272 | 9.51E-05 | 0.014648 | 1.229819 |
| KIF14 | 1.288721 | 1.994163 | 4.036607 | 0.000108 | 0.015252 | 1.119568 |
| EXOSC4 | 1.376929 | 2.877599 | 4.006346 | 0.00012 | 0.015627 | 1.02101 |
| E2F8 | 1.485157 | 1.642102 | 4.007659 | 0.00012 | 0.015627 | 1.025274 |
| PADI4 | 2.076529 | 4.128622 | 3.97609 | 0.000134 | 0.016494 | 0.922986 |
| CENPW | 1.769936 | 2.186653 | 3.97277 | 0.000136 | 0.016568 | 0.912262 |
| OLFM4 | 151.2127 | 138.4045 | 3.966411 | 0.000139 | 0.016634 | 0.89174 |
| ANLN | 1.633764 | 2.01899 | 3.941084 | 0.000152 | 0.017377 | 0.810226 |
| NCAPG | 1.437682 | 1.847495 | 3.897473 | 0.000178 | 0.019016 | 0.670743 |
| UBE2S | 1.1283 | 2.010143 | 3.878367 | 0.00019 | 0.019801 | 0.609983 |
| CCNB1 | 1.498621 | 2.05652 | 3.800005 | 0.000251 | 0.022718 | 0.363035 |
| GINS1 | 1.179171 | 1.75952 | 3.751856 | 0.000297 | 0.02536 | 0.213115 |
| SLC22A4 | -1.71404 | 4.652418 | -3.73771 | 0.000312 | 0.026369 | 0.169323 |
| S100P | 2.749243 | 6.314112 | 3.723808 | 0.000328 | 0.027174 | 0.126428 |
| CKAP4 | 1.945926 | 4.935922 | 3.666289 | 0.0004 | 0.030286 | -0.04984 |
| SQRDL | 1.002014 | 2.790918 | 3.604845 | 0.000493 | 0.034489 | -0.23588 |
| GMNN | 1.028779 | 1.620908 | 3.594495 | 0.000511 | 0.035091 | -0.26698 |
| CDKN3 | 1.298371 | 2.256128 | 3.540673 | 0.000612 | 0.039628 | -0.42765 |
| FCER1G | 1.297229 | 4.202694 | 3.530463 | 0.000634 | 0.040277 | -0.45792 |
| DSC2 | -1.77641 | 2.957786 | -3.52185 | 0.000652 | 0.041 | -0.48339 |
| ZWINT | 1.53315 | 1.924714 | 3.468377 | 0.000779 | 0.045028 | -0.64056 |
| CCNA2 | 1.001536 | 1.726332 | 3.449586 | 0.000829 | 0.046106 | -0.69534 |
| NEXN | 1.021839 | 1.726668 | 3.441045 | 0.000853 | 0.046106 | -0.72017 |
| ATP9A | 1.088746 | 2.796306 | 3.446107 | 0.000839 | 0.046106 | -0.70546 |
| CD24 | 1.85936 | 2.434588 | 3.453191 | 0.000819 | 0.046106 | -0.68485 |
| CTSL | 1.147264 | 2.044918 | 3.435722 | 0.000868 | 0.046421 | -0.73562 |
| PLIN3 | 1.233343 | 3.220398 | 3.424076 | 0.000902 | 0.047246 | -0.76935 |
| CA4 | 4.541004 | 8.295087 | 3.42409 | 0.000902 | 0.047246 | -0.76931 |
| GGH | 1.531007 | 2.842888 | 3.418367 | 0.000919 | 0.047313 | -0.78586 |
| CHIT1 | 2.903221 | 3.63102 | 3.420502 | 0.000913 | 0.047313 | -0.77969 |
| RGL4 | 4.779343 | 8.937969 | 3.409492 | 0.000946 | 0.047785 | -0.81147 |
| MELK | 1.186507 | 1.672102 | 3.402212 | 0.000969 | 0.048093 | -0.83245 |
| FABP5 | 2.207593 | 2.285612 | 3.394252 | 0.000994 | 0.048277 | -0.85534 |
